# Illinois State Medical Society

**Published:** 1854-06

**Authors:** 


THE NORTH-WESTERN

MEDICAL AND SURGICAL JOURNAL.

NEW SERIES.

VOL. III. JUNE, 1854. NO. 6.

### ORIGINAL COMMUNICATIONS.

*Illinois State Medical Society.*

THE Fourth Annual Meeting of the Illinois State Medical Society commenced on Tuesday June 6th, 1854, in the Congregational Church at Lasalle.

The meeting was called to order by the President, Professor DANIEL BRAINARD, at 3 o'clock P. M.

Dr. WELCH, of Lasalle, Chairman of the Commit ee of Arrangements, welcomed the Delegates to the hospitalities of the place in a few appropriate remarks, and then read a list of those delegates who had presented their credentials. About forty members were in attendance, of whom the following had presented their credentials from county societies and local institutions viz:

| | |
|---|---|
| *Esculapian Medical Society,* | DR. SAMUEL THOMPSON. |
| *McLean County Med. Society,* | A. H. LUCE. |
| *Winnebago Co. Med. Society,* | G. P. RANSOM.<br>JOSEPH BLOUNT. |
| *Putnam County* " " | WM. MCKNIGHT. |
| *Lasalle* " " " | O. P. HATHAWAY.<br>JOHN GILLET. |
| *Macon* " " " | S. Y. BALDWIN. |
| *Cook* " " " | H. A. JOHNSON. |
| *Mercy Hospital, Chicago,* | N. S. DAVIS. |

| | |
|---|---|
| *Stark County Medical Society,* | THOS. HALL.<br>T. D. FITCH. |
| *Knox* " " " | G. C. PATTERSON. |
| *Putnam* " " " | E. S. COOPER. |
| *Stark* " " " | —— BODLEY. |
| *Peoria Medical Society,* | J. N. NIGLAS. |
| *Marshall Co. Med. Society.* | C. M. BAKER.<br>JAMES McCOY. |
| *Rush Medical Society,* | D. BRAINARD. |
| *Fulton Co.* | J. C. FITZ. |

On motion of Dr. SAMUEL THOMPSON, of Albion, a committee of one from each County represented, was appointed to recommend candidates for officers of the Society during the coming year.

After a short recess to enable the members from each county to select one of their number to serve on the Committee, it was found to be composed as follows, viz.:

Dr. H. A. JOHNSON, of Cook County.
WM. McKNIGHT, of Putnam "
" J. C. FITZ, of Fulton County.
" J. BLOUNT, of Winnebago "
" J. N. NIGLAS, of Peoria "
" SAM'L THOMPSON, of Esculapian Medical Society.
" J. STOUT, of Lasalle County.
" THOMAS HALL, of Stark "
" JAMES McCOY, of Marshall Co.
" A. H. LUCE, of McLean "
" S. Y. BALDWIN, of Macon "
" G. C. PATTERSON, of Knox "
" T. D. FITCH, of Henry "
" D. W. YOUNG, of Kane "

On motion of Dr. N. S. DAVIS, all members of the Medical profession present and in good standing, were invited to take seats in the meeting as members by invitation.

The next order of business being the election of permanent members, the following gentlemen were proposed and unanimously elected, viz:

| | | |
|---|---|---|
| Dr. R. M. McARTHUR, Ottawa, | proposed by | Dr. N. S. Davis. |
| " D. W. YOUNG, Aurora, | " | " " |
| " ABNER HARD, Ottawa, | " | " " |
| " G. STILLING Winchester | " | by E. S. Cooper. |
| " W. H DAVIS, Lasalle, | " | Dr. Welch. |
| " A. G. LAWTON, Lasalle, | " | " |
| " JOHN HIGGINS, Peru, | " | O. P. Hathaway. |
| " L HALL, Peru, | " | " |
| " J. T. MILLING, Peru, | " | " |
| " W. H. NANCE, Fulton, | " | J. C. Fitz. |
| " E. C. GARDNER, Fulton, | " | " |
| " A. M. JOHNSON. Fulton, | " | " |
| " DEXTER CLARK, Rockford, | " | C. N. Andrews |
| " JAMES CHAMBERS, Peoria, | " | Dr J. N. Niglas. |
| " G. C. WIRTZ, Peru, | " | John Gillett. |
| " J. F. WEEKS, Peru, | " | " |
| " GUY HULETT, Peru, | " | " |
| " JOHN L. FYFIELD, Peru, | " | Thomas Hall. |

On motion of Dr. N. S. Davis, the committee on nominations was instructed to report also a place for holding the next annual meeting of the Society.

The nominating committee than came in with the following report:

*President.*

Dr. C. N. ANDREWS, Rockford.

*Vice Presidents.*

Dr. SAMUEL THOMPSON, Albion,
Dr. THOMAS HALL, Toulon.

*Secretaries.*

Dr. H. A. JOHNSON, Chicago.
Dr. A H. LUCE, Bloomington.

*Treasurer.*

Dr. N. S, DAVIS, Chicago.

The report of the committee was accepted, and the candidates recommended unanimously elected by a vote of the Society. The same committee also reported in favor of holding the next annual meeting at Bloomington, which was also adopted.

The reports of standing committees were then called for, and the chairmen of the committees on Surgery and Practical Medicine were both found absent.

Dr. Thomas Hall, chairman of the committee on Midwifery and the diseases peculiar to women and children, requested that his report might be made the order of business at the commencement of the morning session to-morrow, which request was granted.

Dr. Brainard in retiring from the chair, announced his readiness to comply with the rule requiring from the President an address.

Dr. Welch stated, that arrangements had been made for hearing the address of Dr. Brainard at the afternoon session to-morrow.

The Chairman of the Standing Committee on Drugs and Medicines was absent, and no report.

On motion of Dr Brainard, the President of the Society was directed to appoint the Standing Committees for the ensuing year.

Dr. Davis, of Chicago, expressed his surprise and regret that so many of the Standing Committees for the past year had entirely neglected their duty. He alluded to the fact that members of the Society looked to the reports of the Standing Committees for a large share of the interest and profit of our meetings; and consequently that no man should accept a place, especially as Chairman on such committees, unless he faithfully performs the duty assigned him.

Dr. Brainard remarked that the fault was perhaps more with the profession than the Committees. The latter must depend in a great measure on the former for materials to report on; and he knew that it was sometimes the case that committees failed wholly to get any responses to their application for information.

Dr. S. Thompson, of Albion, offered the following.

*Resolved*, That no man employed or interested in the sale of patent medicines or nostrums either as Druggist or otherwise, can become a member of this society.

After brief remarks from Dr. Brainard, Thompson, Hall, Davis, and others, the resolution was withdrawn on the ground that the present rules and Ethics of Society are sufficient to exclude the class of persons contemplated in that resolution.

The Secretary then presented the reports of the Treasurer and the Committee on Publication, which were accepted and properly referred.

Dr. Davis, of Chicago, moved the following resolution which was adopted, viz.:

*Resolved*, That the Treasurer be instructed to notify immediately the absent members of the Society of the amount of the annual assessment and that the Committee of Publication be directed to withhold from the published list, the names of all such as do not pay the same to the Treasurer before the first of August next.

Notice was given that Dr. N. S. Davis had accepted the invitation of the Committee of Arrangements to address the Society and the public on the Physiological effects of Alcoholic drinks on man, and their value as preventives of disease, this evening at 7½ o'clock.

On motion the Society then adjourned until 9 o'clock to-morrow morning.

In the evening a very respectable audience assembled and listened with diminished interest for an hour and a half, to the address of Dr. Davis. The address was purely scientific and extempore, yet couched in such language as to be readily comprehended by the non-professional hearer.

He showed by a variety of facts and experiments that Alcoholic drinks, instead of being valuable as preventives of disease, are among the most direct and powerful predisposing causes to which man can be subjected.

---

SECOND DAY.

Wednesday, 9 o'clock A. M., the Society was called to order; the President, Dr. C. N. Andrews in the Chair.

The minutes of the yesterday's proceedings were read by the Secretary and approved.

On motion of Dr. N. S. Davis, the amount of the annual assessment on each member for the present year was fixed at $2.

The special order of business being called for, Dr. Thos. Hall, in behalf of the Committe on Prize Essays reported that only one essay had been received by the Committee. That one, however, was possessed of more than ordinary merit, and was well entitled to the premium of $20, donated by Dr. N. S. Davis. The sealed note accompanying the essay being opened, the name of the author was found to be Henry Parker, M. D., of Chicago, Ill.

On the suggestion of Dr. Hall, the essay was referred to the

Committee of Publication, with instructions to permit the author to make certain additions if he wished to do so.

Dr. Thos. Hall, as Chairman of the Committee on Obstetrics, stated that he had prepared no regular report, but with the permission of the Society, would read the report of a case communicated to him by Dr. B. F. Halleck of Vandalia.

Permission was granted, and Dr. Hall read an account of a case of difficult labor, produced apparently by the complete ossification of the bones of the cranium of the child.

The obstruction to labor was so great that the practitioner in attendance attempted to perforate the head.

Dr. Brainard expressed doubts as to the propriety of the operation resorted to, and thought the forceps or other means compatible with the life of the child should have been tried first. He alluded to a case which occurred in his own practice, when the head remained at the superior strait of the pelvis, in which Dr. John Evans of Chicago applied his Obstetrical Extractor and delivered with safety both to mother and *child.*

Dr. Hall related verbally several interesting cases of difficult and complicated labors which had occurred in his own practice. In one case the woman was delivered of two children, one of whom was remarkably deformed and had been so long dead that putrefaction was far advanced and the Liquor Amnii exceedingly offensive; and yet the other child, though much emaciated, was alive and well.

Dr. Hall, also related a case of phlegmasia dolens which he treated successfully with opium to allay pain, with quinine, camphor and oil of turpentine internally, and the application of warm oil of turpentine externally to the affected limb. He stated that he had seen very bad results from treating this disease by antiphlogistic remedies, such as bleeding, cathartics, etc.

Dr. Brainard agreed with Dr. Hall in reference to the treatment of phlegmasia dolens, and stated that most of the cases which had come under his observation had been in *debilitated* patients. He also alluded to several cases of *phlebitis* affecting the illiac and pelvic veins of the male subject, of the same nature as the uterine phlebitis in the female. Two of these cases occurred in connection with hæmorrhoidal tumors in the rectum. Dr. Brainard also alluded to a case of genuine uterine phlebitis which occurred in the practice of another physician. The patient got better for a few days, when the right limb began to swell and gave signs of inflammation of the illiac vein. The attending physician applied a bandage to the affected limb and left the patient without observing any immediately dangerous symptoms. He had scarcely left the house, however, when the patient expired. Dr. Brainard assisted in making a post mortem examination, and found that the inflammation had extended from the uterus into the illiac vein, effusing coagulable lymph on the inner surface of the vessel, which presented the form of *vegetations* projecting into, and more or less obstructing the current of blood. One of these vegetations had apparently been dislodged and carried by the current of blood to the heart, where it was found lodged on the valves and surrounded by a coagulum of blood sufficient to fatally obstruct the circulation.

Dr. E. S. Cooper then read a lengthy paper, entitled, "Walking a primary Element in the Treatment of certain Deformities of the Limbs." — He also exhibited several instruments, invented by himself, for treating club-foot, false anchlylosis of the knee-joint, and coxalgia. The instrument for remedying the deformity of the knee he has used several years with very gratifying results. That devised for treating coxalgia or hip disease is intended to enable the surgeon to effect, not only extension and counter extension, as recommended by Dr. A. March, of Albany, but also the abduction of the upper part of the thigh. He stated that he had not yet had an opportunity of testing fully the utility of the instrument by experience.

On motion of Dr Baldwin, the thanks of the Society were tendered to Dr. Cooper for his communication, and he was requested to condense the same in a manner suitable for the transactions of the Society.

The President then read the following communication viz:

*President of the Illinois State Medical Society.*

DEAR SIR: In conformity with the recommendation of the Committee on Prize Essays, please forward to Henry Parker, M. D., of Chicago, an order on me for the *twenty dollars* offered at our last annual meeting. And please to inform the Society that the same sum is continued at their disposal for prize essays another year. Yours truly

N. S. DAVIS, of Chicago.

Dr. D. Brainard, commended the policy of offering premiums for essays, and also the liberality of the member whose communication has just been read. But he thought it unjust to accept a donation from an individual member, and offer it in the name of the Society. He therefore, moved, that *fifty* dollars be raised by voluntary contributions, and offered by the Society for prize essays during the coming year; the whole to be awarded for one essay, or twenty-five dollars each for two, as the committee on prize essays should judge best. The motion of Dr. Brainard was seconded and adopted by the Society.

The election of permanent members was again taken up and the following gentlemen proposed and duly elected, viz.:

| | | | | |
|---|---|---|---|---|
| Dr. | J. F. WORRELL, | Bloomington, | proposed | by Dr. S. W. NOBLE. |
| " | WM CROMWELL | do | do | do do |
| " | G. W. STIPP | do | do | do do |
| " | KELLER, | Decatur, | do | S. Y. BALDWIN. |
| " | TROWBRIDGE, | Decatur | do | do |
| " | KINKADE, | do | do | do |
| " | T. LEEDS | of Mt Pulaski | do | S. W. NOBLE. |
| " | S. L. CRAIG | of Delta | do | do |
| " | A. A. LOVEJOY | of Aurora | do | D. W. YOUNG. |
| " | P. A ALLAIRE | do | do | do |
| " | P. KIRWIN | of Ottowa | do | J. STOUT. |
| " | C. HARD | do | do | do |
| " | T. HEY | do | do | do |
| " | H. SMITH | of Chicago | do | N. S DAVIS. |
| " | J. C. MORFITT | do | do | D. BRAINARD, |

On motion the Society adjourned to 2 o'clock P. M.

---

### Afternoon Session.

At 2 o'clock P. M., the President, Dr. Andrews, took the Chair and called the meeting to order.

The Standing Committees for the ensuing year were then announced as follows, viz:

*Committee on Surgery.*

Dr. FREER, of Chicago.
" JAMES BLOUNT, of Oregon.
" J LEEDS, of Mt. Pulaski.

*Committee on Practical Medicine.*

Dr. EDWARD ROE, of Bloomington.
" S. W. NOBLE, of Leroy.
" S. Y. BALDWIN, of Decatur.

*Committee on Obstetrics.*

Dr. J L. HAMILTON, of Peoria.
" C. M. BAKER, of Henry.
" W. W. WELCH, of Lasalle.

*Committee on Drugs and Medicines.*

Dr. H. A. JOHNSON, of Chicago.
" G. P. RANSOM, of Roscoe.
" JOSEPH STOUT, of Ottawa.

*Committee of Arrangements.*

Dr. A. H. LUCE, of Bloomington.
" EDWARD ROE, do
" J. F. WORRELL, do

The President was authorized to fill any vacancies that might occurs in the Standing Committees.

Dr. D. Brainard, the Ex-President, then delivered his address; the subject of which was the local treatment of poisoned wounds The effects of certain poisons on birds, and the efficacy of iodine in counteracting these effects, were illustrated by direct experiments performed before the Society. The paper was an extremely inte[illegible] esting and valuable one ; but as it will appear in full in the printed volume of transactions, we refrain from giving any analysis of it here.

On motion of Dr. S. Thompson the thanks of the Society were tendered to Dr. Brainard for his very interesting address, and a copy of the same requested for publication in the transactions.

Dr. J. O. Harris, of Ottawa, presented his credentials as delegate from Lasalle County Medical Society and took his seat in the Society.

The following was offered by Dr. S. Thompson. viz:

*Resolved*, That a Committee of —— be appointed to obtain the names of all the regular practitioners of medicine in the State, with their address; and also a list of all the medical societies at p e ent existing

Dr. N S. Davis offered the following as a substitute which was adopted, viz:

*Resolved*, That a Committee of three be appointed to obtain the names of the regular members of the profession throughout the State, with their address; and to use their influence to procure the formation of County Medical Societies in all those counties where none exist.

The President appointed Dr Samuel Thompson, of Albion, Dr. H. A Johnson, of Chicago and Dr. A. B Chambers, of Toulon, said committee.

Dr S. Thompson offered the following which was adopted, viz:

*Resolved*, That members of this Society before pro osing any one as a canditate for election as permanent member, should be careful to ascertain whether such candidate is in any way connected with, or interested in, the sale of patent medicines and nostrums; such persons being incompetent to become members. See section 4. chapters 2 and 3 of code of Ethics.

Dr. N. S. Davis offered the following which was adopted:

*Resolved*, That the President appoint a committee of three to receive and ex mine all essays that may be presented for the prize or prizes offered by this Society. And that writers be allowed to choose their own subjects, it being understood that all essays must be sent to the chairman of the committee on or before the 1st of May 1855.

The President appointed Drs N. S. Davis, of Chicago, Edward Dickinson, of Peoria, and J. O. Harris, of Ottawa, the Committee on Prize Essays.

Dr. D. Brainard moved, that Delegates to the American Medical Association be appointed by the President of the Society.

Dr. Baldwin moved as an amendment, that the Society now proceed to the appointment of said delegates; which was adopted and the following members elected by vote of the Society, viz:

Dr. D Brainard, of Chicago; Dr. A. H. Luce, of Bloomington;

Dr. E S. Cooper, of Peoria; Dr. Samuel Thompson, of Albion; Dr. C. N. Andrews, of Rockford; Dr S. Y Baldwin of Decature; Dr. S W. Noble, of Leroy; Dr. J. Higgins, of Peru; Dr. W. W. Welch, of Lasalle; Dr. H A Johnson, of Chicago; Dr. J. C. Fitz of Fulton; Dr. J. W. Spalding, of Galesburg.

On motion of Dr. Brainard, it was resolved, that those delegates who may attend the next meeting of the American Medical Association, be authorized to fill any vacancies that may be found to exist in their body

Dr. S. Thompson offered the following as an amendment to the constitution, which was laid on the table until the next annual meeting, viz :

*Resolved*, That respected members of this Society who may remove from this State, and distinguished members of the profession in other States, may be elected honorary members of this Society by a unanimous vote.

On motion of Dr. S. W. Noble, the committee on publication was authorized to re publish in the next volume of transactions, the constitution and code of Ethics of this Society.

Dr. D. Brainard, offered the following which was unanimously adopted, viz :

*Resolved*, That the Committee of Arrangements of this Society be instructed to omit preparing or having prepared by the local profession, any general *entertainment* at the annual meetings of the Society.

On motion of Dr. Baldwin the thanks of the Society were tendered to the Congregational Church of the city of Lasalle, for the use of their church edifice ; and also to the Committee of Arrangements and other officers of the Society for the judicious manner in which they have discharged their duties.

On motion the Society adjourned *sine die*.

H. A JOHNSON,

A. H. LUCE. } *Secretaries.*

